# Acute kidney injury and postoperative delirium

**DOI:** 10.7555/JBR.39.20250354

**Published:** 2026-09-28

**Authors:** Linyi Yang, Chen Li, Charandip Sandhu, Hong Liu, Chen Chen

**Affiliations:** 1 Department of Anesthesiology, the Third Affiliated Hospital of Soochow University, Changzhou, Jiangsu 213000, China; 2 Department of Anesthesiology and Pain Medicine, University of California Davis Health, Sacramento, CA 95817, USA

**Keywords:** acute kidney injury, delirium, perioperative neurocognitive dysfunction, postoperative complications

## Abstract

Postoperative acute kidney injury (AKI) and postoperative delirium (POD) are two of the most common complications after surgery. It is essential to understand their common mechanisms not only to prevent and predict the occurrence of these complications, but also to guide perioperative management and improve surgical outcomes. This review summarizes the recent literature focused on these postoperative complications and explores the relationship between AKI and POD. Clinical studies suggest that AKI and POD may coexist or occur sequentially during the perioperative period, particularly in susceptible patients undergoing major surgery. Some research findings indicate that the brain and the kidney exhibit bidirectional communication that is affected by systemic inflammation, drug accumulation, uremic toxins, vascular endothelial injury, and fluid volume redistribution during the perioperative period. AKI and POD may share pathophysiological mechanisms involving multiple interacting pathways, leading to impairment of renal and cerebral functions.

## Introduction

More than 10% of surgical patients experience major postoperative complications, leading to increased healthcare costs, prolonged hospital stays, and increased morbidity, including but not limited to acute kidney injury (AKI), postoperative delirium (POD), deep venous thrombosis, pulmonary embolism, pneumonia, myocardial infarction, infections, and blood clots. Among these, postoperative AKI and POD are two of the most common complications after surgery^[1]^. Numerous studies have been conducted to predict these complications based on perioperative comorbidities and risk factors to help reduce their incidence through early diagnosis and interventions^[2–8]^. In clinical practice, we have observed that AKI and POD frequently occur simultaneously or sequentially in postoperative patients. AKI and POD are both important clinical problems associated with adverse short- and long-term outcomes^[9–10]^, but they are often underestimated because of their subclinical manifestations and different diagnostic criteria^[11–12]^. Understanding the common factors and mechanisms underlying AKI and POD may help us provide better care for patients in this susceptible population.

Literature from January 2015 to December 2024 was retrieved from PubMed, Web of Science, and Google Scholar using the keywords acute kidney injury, delirium, perioperative neurocognitive dysfunction, and postoperative complications. Relevant articles focusing on major postoperative complications and their potential shared mechanisms were reviewed.

### Acute kidney injury

AKI following major surgery is a significant postoperative complication that is closely linked to higher mortality, the occurrence of other complications, prolonged hospitalization, and the onset of chronic kidney disease (CKD), leading to substantial healthcare costs and societal challenges^[9,13]^. Given that AKI occurs after a variety of surgeries, consensus statements have focused on AKI following both cardiac and noncardiac surgical procedures in the perioperative period^[14–15]^. The Kidney Disease: Improving Global Outcomes (KDIGO) criteria currently used to define AKI in most studies are based on changes in baseline serum creatinine (SCr) and urine output. However, the unavailability of baseline SCr in emergency surgery and the administration of different fluid regimens that cause volume changes may lead to difficulties in diagnosing subclinical AKI, thereby leading to an underestimation of its incidence^[16]^. Studies have focused on identifying the precipitating factors to help predict and reduce the occurrence of AKI since there is no efficient treatment^[5,17–18]^.

### Postoperative delirium

As the population ages, the susceptibility of elderly patients to anesthesia has led to an increasing incidence of perioperative neurocognitive disorders (PND), which encompass cognitive disturbances occurring before or after surgery, including postoperative delirium and longer-lasting postoperative neurocognitive impairment^[19–20]^. In elderly patients, the diagnosis of PND is associated with a significant increase in healthcare costs in the year following surgery, demonstrating the importance of PND as a worthy target for value-based healthcare improvement.

POD is one of the most common forms of PND, characterized by acute and fluctuating impairment in attention, memory, perception, and consciousness occurring in the early postoperative period and lasting a few days^[21]^. Its incidence varies widely according to patient characteristics, type of surgery, and diagnostic methods, with particularly high rates reported in older and other high-risk surgical populations^[22]^. Delirium may manifest in hyperactive, hypoactive, and mixed subtypes, of which the hypoactive and the mixed subtypes are more difficult to detect, resulting in an underestimation of the incidence^[12]^. Patients with certain preoperative risk factors, including male sex, age over 65, preexisting cognitive impairment, and medical conditions like diabetes, renal insufficiency, congestive heart failure, cerebrovascular accident, and transient ischemic attack, are more likely to develop POD^[23]^. The mechanisms leading to delirium, however, remain poorly understood^[24]^. The pathophysiology of delirium broadly consists of direct central nervous system insults and aberrant stress responses^[25–30]^.

## Common pathophysiological pathways of AKI and POD

Although the relationships between AKI and POD are not fully understood, some common pathways and pathophysiological changes in the perioperative period may cause them to occur simultaneously^[31]^. The association between AKI and POD is not a simple causal relationship; rather, it is a complex network composed of multiple interacting mechanisms. Understanding these mechanisms may help guide treatment and optimize healthcare for patients undergoing major surgeries (***Table 1***).

**Table 1:** Common risk factors for acute kidney injury and postoperative delirium

Categories	Risk factors	Mechanisms
Patient baseline vulnerability	Advanced age	Impaired organ autoregulation; chronic comorbidities
	Chronic kidney disease	Diminished renal function; accumulation of uremic guanidino compounds
	Cerebrovascular disease	Microvascular damage
	Cardiovascular disease	Insufficient cardiac output; hypotension and hypoperfusion
	Diabetes	Vascular endothelial injury in renal and cerebral vessels
Surgery-related factors	Systemic inflammation	Trauma-induced release of pro-inflammatory mediators; renal tubular injury; increased permeability of the blood-brain barrier and neuroinflammation
	Perioperative hypotension and hypoperfusion	Ischemia and hypoxia; oxidative stress
	Fluid overload	Venous congestion and increased renal interstitial pressure
	Vasopressors	Decreased perfusion in the kidney and brain
Drugs	Nephrotoxic drugs	Direct renal injury; accumulation of uremic toxins
	Anticholinergic agents	Disruption of neurotransmitter metabolism; inhibition of the cholinergic anti-inflammatory pathway

### Systemic inflammation

Ischemia- and hypoxia-induced renal tubular injury, surgical trauma, and nephrotoxins can all lead to postoperative AKI. All these injuries may involve the same inflammatory pathway. Injured cells release damage-associated molecular patterns (DAMPs), including myoglobin, uric acid, high mobility group box 1 (HMGB1), ATP, and DNA fragments, into the circulation during surgery, which may be the initial trigger in many perioperative complications. DAMPs interact with pattern recognition receptors, such as the classical inflammatory signal receptor Toll-like receptor 4 (TLR4), on bone-marrow-derived macrophages (BMDMs) in the circulation, and mediate downstream inflammatory responses through the nuclear factor kappa B (NF-κB) pathway to release pro-inflammatory cytokines such as tumor necrosis factor alpha (TNF-α), interleukin-1 beta (IL-1β), IL-6, and IL-18, as well as chemokines^[32–33]^. Studies have reported notable elevations in inflammatory markers in the central nervous system following anesthesia and surgery^[34–37]^. In the brain, the chemokine monocyte chemoattractant protein-1 (MCP-1) binds to the TLR4 receptor on the microvascular endothelial cells and pericytes, increasing the permeability of the blood-brain barrier (BBB), recruiting macrophages into the central nervous system, and activating resident microglia. BMDMs and neurotoxins migrate to the hippocampus through the compromised BBB and activate microglial cells to release pro-inflammatory cytokines, such as IL-1β and IL-6, potentially disrupting synaptic plasticity and neuronal function^[38]^. The neuroinflammation and activated microglia observed following surgery and anesthesia are considered important mechanisms leading to POD.

A similar mechanism occurs in AKI because of the activation of the inflammatory system triggered by surgical stimulation and trauma^[39–40]^. In patients undergoing major surgery without pre-existing AKI, inflammatory mediators in the circulation are initially recognized by receptors on dendritic cells and renal cells in the kidney. These intrarenal immune cells respond to DAMPs by actively secreting cytokines such as IL-1, IL-6, and MCP-1, which migrate to nearby blood vessels and recruit BMDMs from the circulation to the kidney, and these recruited non-specific macrophages can also induce kidney injury^[41]^. The REALAKI study, a sub-analysis of the REAnimation Low Immune Status Marker (REALISM) cohort, reported a significant increase in inflammatory markers in the blood as well as alterations in the innate and adaptive immune responses in AKI patients following different types of injuries, including surgery^[42]^. In a multicenter cohort study that included 960 individuals, increased concentrations of postoperative pro-inflammatory and anti-inflammatory cytokines (IL-6 and IL-10) were associated with an increased risk of AKI after cardiac surgery, suggesting a correlation between the systemic inflammatory response and AKI^[43]^ (***Fig. 1***). AKI may increase susceptibility to POD through systemic inflammation, cerebral microvascular dysfunction and disruption of BBB integrity^[42,44–45]^. Injured kidney cells also induce the release of a range of pro-inflammatory cytokines into the circulation and affect remote organs such as the brain, and systemic inflammation potentially plays an important role in the pathogenesis of delirium^[44]^.

**Figure 1:**
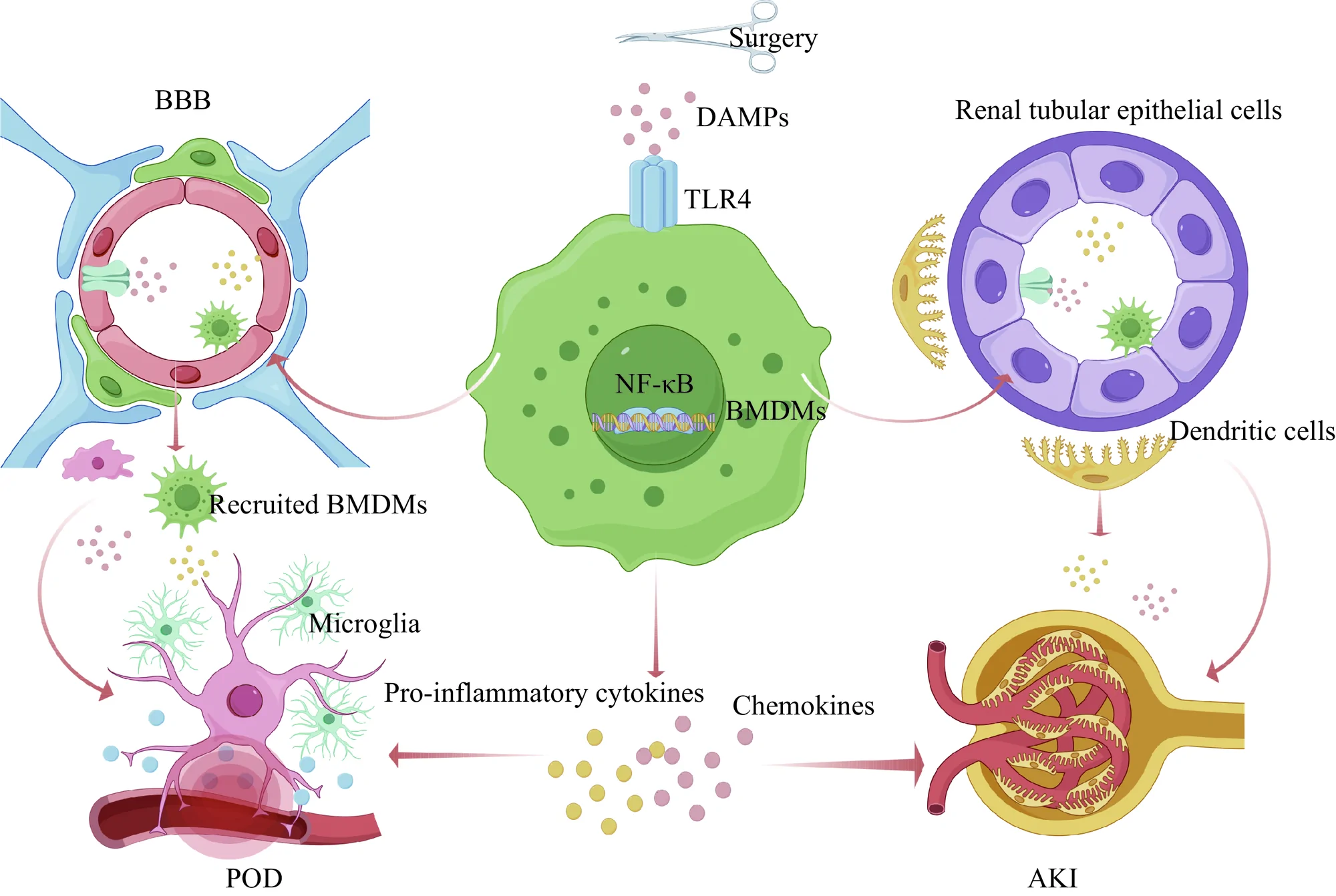
Schematic illustration of systemic inflammation in surgery. DAMPs released by surgery interact with TLR4 on BMDMs and activate the NF-κB inflammatory pathway to release pro-inflammatory cytokines and chemokines, which affect resident immune cells, including microglia in the brain and dendritic cells in the kidney, thereby contributing to organ dysfunction. Abbreviations: AKI, acute kidney injury; BBB, blood-brain barrier; BMDMs, bone marrow-derived macrophages; DAMPs, damage-associated molecular patterns; POD, postoperative delirium; TLR4, Toll-like receptor 4.

These inflammatory mediators can affect the BBB directly or indirectly *via* neuroendocrine pathways. Metabolic disorders of neurotransmitters may disrupt complex brain functions and lead to coma, hyperexcitability, and other symptoms. Notable changes in neurotransmitter levels have been detected in animal AKI models, including decreased dopamine turnover in the striatum, mesencephalon, and hypothalamus, which can substantially affect learning, memory, anxiety, and depression^[46]^. Maldonado and colleagues found that a systemic inflammatory response can cause neurotransmitter disturbances, such as changes in dopamine, acetylcholine, and serotonin levels, which are related to postoperative delirium^[47]^. The authors also suggest that modulating these neurotransmitter levels may help to prevent and treat delirium. The accumulation of toxins resulting from renal insufficiency may also contribute to dysregulated metabolism of neurotransmitters such as acetylcholine, dopamine, glutamate, and γ-aminobutyric acid (GABA), potentially inducing or exacerbating delirium.

In addition to the systemic effects of inflammatory factors, the cholinergic anti-inflammatory pathway may also play an important role in the co-occurrence of AKI and POD. This pathway may protect the kidney from ischemia/reperfusion injury by suppressing pro-inflammatory cytokines in the kidney^[48]^. Studies suggest that dexmedetomidine, an adjuvant used in general anesthesia, leads to stable perioperative cholinesterase activity and a decrease in neuroinflammation through the cholinergic anti-inflammatory pathway, and that the cholinesterase activity, together with other factors, is related to the development of POD^[49–50]^.

### Intraoperative hypotension and hypoperfusion

Adequate blood flow is critical for organs like the kidney and the brain to maintain normal glomerular filtration rate (GFR) and cerebral metabolism, respectively^[16]^. It has been reported that intraoperative hypotension (IOH) is associated with increased 30-day mortality and serious postoperative complications, including AKI and POD, given that the brain and kidney are both sensitive to hypotension-induced injury^[51–55]^. It has been suggested that maintaining a higher mean arterial pressure (MAP) during cardiopulmonary bypass (CPB) increases intraoperative and postoperative diuresis and reduces the release of renin and the incidence of POD in patients undergoing cardiac surgery^[56]^. There is no clear consensus on the standard criteria for IOH. Different definitions of hypotension have been used, including a systolic blood pressure (SBP) less than 80 mmHg, MAP less than 65 mmHg, or blood pressure 20% below the baseline^[52–53]^. Hypovolemia, anesthetic-induced vasodilation, positive pressure ventilation, and cardiac dysfunction contribute to hypotension. In certain laparoscopic abdominal procedures, increased abdominal pressures may cause congestion and low arterial perfusion pressure, leading to organ injury^[57]^.

Although both the kidney and the brain autoregulate over a wide range of blood pressures, this important physiological function is ineffective in some susceptible patient populations, such as the elderly and those taking angiotensin-converting enzyme inhibitors/angiotensin receptor blockers (ACEI/ARBs)^[56,58]^. Guiding blood pressure management based on absolute thresholds may be inadequate to protect organ function^[59]^, and further interventions may be required in daily clinical practice. Research suggests that transcranial Doppler monitoring of the middle cerebral arteries during CPB to optimize MAP, by maintaining it above the lower limit of cerebral autoregulation, may reduce the incidence of delirium after cardiac surgery by 45%^[60]^. This finding implicates hypotension as a contributor to delirium.

### Fluids and vasopressors

Fluids, vasopressors, and inotropes are typically administered to treat hypotension based on the potential causes^[57]^. However, the treatment for hypotension itself can also lead to brain and kidney injury^[16]^. Often, volume resuscitation is the first choice to stabilize hemodynamics and organ perfusion for patients in the perioperative period. However, insensitivity to volume resuscitation in some pathological processes is likely to result in volume overload and tissue edema^[61–62]^. Venous congestion caused by volume overload, increased renal interstitial pressure, and decreased renal blood flow and GFR have been proposed as contributors to AKI^[60,63]^. Zarbock and colleagues found that approximately one in five patients developed AKI after major surgery, and that patients with AKI typically received more fluids and more vasopressors, including norepinephrine, dobutamine, epinephrine, and vasopressin^[64]^. It has also been suggested that cerebral edema is commonly seen in patients with delirium^[65]^. Vasopressors can also produce adverse effects due to vasoconstriction affecting the blood supply to organs. Ma and colleagues reported that intraoperative phenylephrine use was associated with a higher incidence of postoperative delirium than ephedrine, potentially due to differences in cerebral blood perfusion; other vasoactive drugs, such as vasopressin, were associated with fewer intensive care unit admissions and a lower incidence of AKI and atrial fibrillation than norepinephrine^[66–67]^. These results suggest that the dose and type of fluid and vasoactive agents should be considered based on different pharmacological mechanisms when administered to severely ill patients to reduce the impact on organs such as the kidney and the brain. However, these observational associations may partly reflect greater illness severity and confounding by indication rather than direct harmful effects of the interventions themselves.

### Accumulation of drugs and nephrotoxins

Many medications, such as sedatives, analgesics, antibiotics, nonsteroidal anti-inflammatory drugs (NSAIDs), and ACEI/ARBs, are used during the perioperative period, but there are few studies on the pharmacokinetics of these drugs in patients with AKI^[31]^. It has been reported that the direct neurotoxic effect of AKI is associated with the accumulation of uremic guanidino compounds (*e.g.*, creatinine, guanidine, guanidinosuccinic acid, and methylguanidine), which contribute to the pathogenesis of delirium through the inhibition of γ-aminobutyric acid receptors and activation of N-methyl-d-aspartate (NMDA) receptors^[68]^. In addition to the accumulation of endogenous toxins, exogenous compounds such as sedatives and opioids may also contribute to the development of POD. Drug pharmacokinetics and exposure may become less predictable because of altered renal clearance and metabolism in patients with AKI^[69]^. The disruption of the BBB permeability allows drugs, metabolites, toxins, and cytokines to penetrate the brain, potentially leading to brain dysfunction and the occurrence of POD. The mechanism of drug-induced delirium in the context of AKI, such as the increased neurotoxicity of the antimicrobial cefepime, has been discussed^[45]^. Siew and colleagues reported that in patients with AKI, renal replacement therapy may facilitate the clearance of certain drugs and metabolites associated with neurotoxicity; however, whether this directly reduces the risk of delirium remains uncertain^[70]^.

Both AKI and POD, two of the most common postoperative complications, can serve as critical indicators for risk prediction and mutual identification. Many high-risk factors for AKI (such as advanced age, diabetes, hypertension, atherosclerosis, and heart failure) are also significant risk factors for POD. These comorbidities could potentially affect patients' cerebral and renal functions and render them more vulnerable to perioperative stress, such as surgical trauma. Patients with these risk factors should undergo early screening for AKI and delirium to avoid underdiagnosis of subclinical cases. Controlling the systemic inflammatory response during the perioperative period could be beneficial for protecting the functions of important organs. Individualized goal-directed fluid administration may be an effective strategy to avoid both volume overload and hypovolemia and to prevent perioperative AKI and delirium. Maintaining hemodynamic stability and ensuring tissue perfusion pressure are also crucial focuses in perioperative management. Although intraoperative hypotension has several definitions, such as a 20% decrease in systolic blood pressure from the baseline and a MAP < 65 mmHg, there is a consensus statement that even brief durations of systolic arterial pressure (SAP) < 100 mmHg and MAP < 60–70 mmHg are associated with AKI in non-cardiac surgery^[71]^. Both absolute and relative thresholds indicate that the injury is a function of the hypotension severity and duration. A study involving 316717 surgical patients concluded that in non-cardiac surgeries, a MAP lower than 55 mmHg is associated with an increased incidence of postoperative delirium^[72]^. Another study indicates that intraoperative hypotension with a MAP lower than 65 mmHg lasting for 5 minutes can increase the incidence of postoperative delirium in elderly patients after thoracic surgery, suggesting that timely correction of intraoperative hypotension can reduce the risk of postoperative delirium^[73]^. The differences in the above absolute thresholds may be attributed to variations in the blood flow autoregulatory functions of the kidney and the brain, as well as differences in organ vulnerability among the research subjects.

Dexmedetomidine has been investigated for its potential renal- and neuroprotective effects in the perioperative setting. Meanwhile, potentially nephrotoxic medications should be avoided or minimized when clinically feasible, while fluid and vasoactive therapies should be individualized to maintain adequate organ perfusion and minimize the risks associated with both hypoperfusion and fluid overload. For example, cefepime is a fourth-generation antibiotic that is widely and safely used in clinical practice. However, it has been reported to cause neurotoxicity in patients with preexisting decreased renal function^[74]^. In patients with renal failure, the concentration of cefepime in cerebrospinal fluid may increase because of reduced renal elimination of the drug and the increased permeability of the BBB. Therefore, cefepime dosing should be adjusted according to renal function, and concomitant nephrotoxic exposure should be minimized whenever clinically feasible. In hip fracture patients with renal impairment, the increased use of intraoperative opioids significantly increases the risk of delirium on the second postoperative day, since renal impairment affects opioid pharmacokinetics and the removal of their metabolites^[75–76]^. Meperidine, an opioid analgesic, is recommended to be avoided as much as possible in elderly patients with more vulnerable central nervous systems because of its side effects including anxiety, delirium, and seizures^[77]^. Normeperidine, the neurotoxic metabolite of meperidine, is excreted by the kidney and has a longer half-life (15–30 h) than meperidine (2–5 h). It tends to accumulate in patients with renal dysfunction and is considered to be associated with delirium and seizures. Greater caution is warranted when combination drug therapy is used in patients with impaired renal function or pre-existing cerebrovascular disease.

Healthcare providers should also maintain vigilance for unrecognized POD, particularly hypoactive delirium, through implementing appropriate diagnostic screening, addressing reversible risk factors, and starting cognitive rehabilitation training at an early stage when postoperative AKI occurs. By conceptualizing the kidney and brain as an interconnected system, proactive prevention and early screening of postoperative AKI become essential strategies not only for renal protection but also for preserving central nervous system function and improving long-term patient outcomes.

## Conclusions

Surgical outcomes can be affected by intraoperative events, such as systemic inflammation, hemodynamic instability, fluid and electrolyte imbalance, and the accumulation of drugs and toxins, all of which could contribute to the occurrence of POD and AKI. Current evidence supports substantial overlap in the risk factors and pathophysiological pathways of postoperative AKI and POD, but does not establish a simple causal relationship between them. Future prospective and mechanistic studies are needed to define the directionality and temporal sequence of kidney-brain crosstalk and determine whether interventions targeting shared pathways can simultaneously reduce the risks of AKI and POD.
